# Correction: Distinct neurexin-cerebellin complexes control AMPA- and NMDA-receptor responses in a circuit-dependent manner

**DOI:** 10.7554/eLife.94305

**Published:** 2023-11-09

**Authors:** Jinye Dai, Kif Liakath-Ali, Samantha Rose Golf, Thomas C Südhof

**Keywords:** Mouse

Dai J, Liakath-Ali K, Golf SR, Südhof TC. 2022. Distinct neurexin-cerebellin complexes control AMPA- and NMDA-receptor responses in a circuit-dependent manner. *eLife* **11**:e78649. doi: 10.7554/eLife.78649.

Published 7 October 2022

We were alerted to isolated errors in the source data files of Figure 1G and 5C by helpful comments on PubPeer and upon reanalysis of the data noticed additional isolated errors in the source data files of Figure 1B and 1F that were not noted on PubPeer. These errors were caused by copy-paste errors during the assembly of the final Excel files for publication. Additionally, we identified one error in the Figure 1—figure supplement 1 that arose during calculations of the coefficient of variation using Igor software. Since the apparent coefficient of variation is dependent on the stimulus intensities, we have systematically re-analysed all conditions to determine the average coefficient of variance as presented in the corrected dataset. None of the corrections change the conclusions of the paper. We sincerely apologize for these errors.

The specific errors and needed corrections are described below:

Figure 1B—Source data 1: In the source data file of Figure 1B, the EPSC amplitude values for the 100 μA stimulation intensity of cell 3 and cell 10 in the “Cbln2^+/+^” condition were mistakenly copied from a previously analysed dataset that used different EPSC trace selection criteria and methods. The original and corrected values are shown below. This change does not change the figure or the conclusion. The source data file has been updated accordingly.

Corrected values for the data in Figure 1B in Figure 1—Source data 1:

“Cbln2^+/+^” condition

Stimulus Intensity: 100 μA.

Cell 3 and cell 10 (pA): 85.332 and 69.236.

Original values for the data in Figure 1B in Figure 1—Source data 1:

“Cbln2^+/+^” condition

Stimulus Intensity: 100 μA.

Cell 3 and cell 10 (pA): 103.867 and 85.262.

Figure 1F—Source data 1: In the source data file of Figure 1F, the PPRs values of cell 6 in the “Cbln2^+/+^” condition were erroneously copy-pasted from cell 5 of the same condition during assembly of the final Excel files for publication. The original and corrected values are shown below. This change does not alter the figure or the conclusion. In addition, the labels (Cbln2^+/+^ and Cbln2^-/-^) of Figure 1F in the source data were mislabelled and are now corrected. The source data file has been updated accordingly.

Corrected values for the data in Figure 1F in Figure 1—Source data 1:

“Cbln2^+/+^” condition

PPR before and PPR after

Cell 6 (PPR): 1.498 and 1.213.

Original values for the data in Figure 1F in Figure 1—Source data 1:

“Cbln2^+/+^” condition

PPR before and PPR after

Cell 6 (PPR): 1.646 and 1.390.

Figure 1G—Source Data 1: In the source data file of Figure 1G, the EPSC amplitude values for the 100 μA stimulation intensity of cells 1, 2, 3, 7, 8, 13 in the “Cbln2^+/+^” condition were mistakenly copy-pasted from a previously analysed dataset that used different EPSC trace selection criteria and methods. The original and corrected values are shown below. This change does not change the figure or the conclusion. The source data file has been updated accordingly.

Corrected values for the data in Figure 1G in Figure 1—Source data 1:

“Cbln2^+/+^” condition

Stimulus Intensity: 100 μA.

cells 1, 2, 3, 7, 8, 13 (pA): 98.573, 143.800, 134.823, 133.315, 85.603, and 218.671.

Original values for the data in Figure 1G in Figure 1—Source data 1:

“Cbln2^+/+^” condition

Stimulus Intensity: 100 μA.

cells 1, 2, 3, 7, 8, 13 (pA): 127.587, 137.628, 130.657, 160.723, 91.116, and 212.394.

Figure 5C—Source data 1: In the source data file of Figure 5C, the data for cell18 of the “Cbln12^dcko^” condition were erroneously copy-pasted from cell 17 of the Cbln12^f/f^ condition during assembly of the final Excel files for publication. The original and corrected values are shown below. This change does not alter the figure or the conclusion. The source data file has been updated accordingly.

Corrected values for the data in Figure 5C in Figure 5—Source data 1:

“Cbln12^dcko^” condition

Interstimulus interval (ms) 20, 80, 200, 800, 2000.

cell 18 (PPR): 2.621, 1.646, 1.197, 1.036, 1.042.

Original values for the data in Figure 5C in Figure 5—Source data 1:

“Cbln12^dcko^” condition

Interstimulus interval (ms) 20, 80, 200, 800, 2000.

cell 18 (PPR): 2.419, 1.513, 1.329, 1.202, 1.055.

Figure 1—figure supplement 1: Due to calculation errors when we used Igor software, we have conducted a comprehensive analysis of the coefficients of variation for each cell at all 7 stimulation intensities. We then calculated the average coefficient of variation for each cell and plotted them in the corrected figure and dataset. This correction does not alter the conclusion. The source data file has been updated accordingly.

We show below the original and corrected versions of Figure 1—figure supplement 1.

Corrected Figure 1—figure supplement 1:

**Figure 1:**
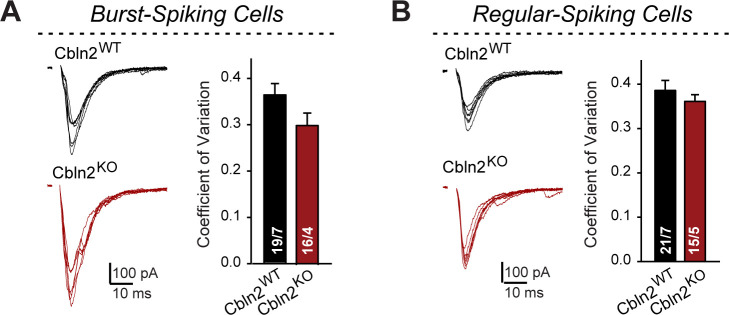


Original Figure 1—figure supplement 1:

**Figure 2:**
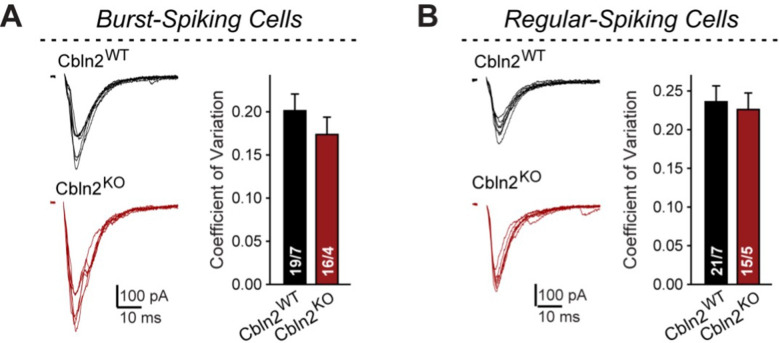


The corresponding figure legend has been corrected accordingly.

Corrected Figure 1—figure supplement 1 legend

**Constitutive *Cbln2* deletion does not alter the coefficient of variation of AMPAR-EPSCs in burst- and regular-spiking subiculum neurons (corresponding to** Figure 1).

(**A & B**) Left, sample traces of evoked AMPAR-EPSCs with 100 μA stimulus intensity (data from Figure 1B and G); right, summary graph of the average coefficient of variation for AMPAR-EPSCs across all 7 conditions of 10–100 μA stimulus intensity. Data are means ± SEM. Number of neurons/mice are indicated in bars. Statistical significance was assessed by unpaired two-tailed t-test revealed no significant differences.

Original Figure 1— figure supplement 1 legend

**Constitutive *Cbln2* deletion does not alter the coefficient of variation of AMPAR-EPSCs in burst- and regular-spiking subiculum neurons (corresponding to** Figure 1).

(**A & B**) Left, sample traces of evoked AMPAR-EPSCs with 100 μA stimulus intensity (data from Figure 1B and G); right, summary graph of the coefficient of variation of AMPAR-EPSCs. Data are means ± SEM. Number of neurons/mice are indicated in bars. Statistical significance was assessed by unpaired two-tailed t-test revealed no significant differences.

We show below the original and corrected source data.

Corrected Figure 1—figure supplement 1—source data 1

**Table:** 

Figure 1—figure supplement 1A		Figure 1—figure supplement 1B	
p *= 0.07833*		p *= 0.40781*	
Cbln2^+/+^	Cbln2^-/-^	Cbln2^+/+^	Cbln2^-/-^
0.2833	0.3185	0.5104	0.3789
0.4134	0.2352	0.4665	0.3634
0.5917	0.5013	0.4338	0.3202
0.2504	0.1963	0.3236	0.3130
0.5846	0.3346	0.4955	0.4048
0.4409	0.2053	0.5520	0.4390
0.2259	0.3073	0.3346	0.3038
0.3869	0.3518	0.4125	0.3434
0.2910	0.2657	0.3000	0.4094
0.5307	0.4345	0.3939	0.4383
0.3442	0.4862	0.3081	0.2582
0.2760	0.1527	0.2288	0.4407
0.3116	0.2465	0.3103	0.3328
0.3440	0.2705	0.5144	0.2869
0.3895	0.3254	0.2305	0.3869
0.2695	0.1408	0.3682	
0.3506		0.4349	
0.3370		0.3230	
0.3038		0.2786	
		0.3182	
		0.5710	

Original Figure 1—figure supplement 1—source data 1

**Table:** 

Figure 1—figure supplement 1A		Figure 1—figure supplement 1B	
p *= 0.33135*		p *= 0.74461*	
Cbln2^+/+^	Cbln2^-/-^	Cbln2^+/+^	Cbln2^-/-^
0.14246	0.18763	0.3498	0.2316
0.22996	0.05544	0.1558	0.1605
0.21689	0.16510	0.2116	0.0930
0.12276	0.06516	0.1425	0.3363
0.41422	0.13429	0.3074	0.2483
0.19480	0.12684	0.2259	0.3407
0.10011	0.14512	0.1953	0.2588
0.26752	0.11027	0.2267	0.1340
0.16355	0.13217	0.1712	0.3068
0.11085	0.21654	0.2862	0.2219
0.13957	0.35038	0.2567	0.1178
0.12711	0.13516	0.1576	0.2724
0.16094	0.16772	0.0469	0.3329
0.18185	0.29314	0.4052	0.1454
0.35577	0.27086	0.1677	0.1879
0.24666	0.22378	0.2241	
0.28587		0.4712	
0.20921		0.2606	
0.15443		0.2892	
		0.2066	
		0.1959	

The article has been corrected accordingly.

