# Supplementary material for: Distinct neurexin-cerebellin complexes control AMPA- and NMDA-receptor responses in a circuit-dependent manner
Source: eLife. 2022 Oct 7;11:e78649. doi: 10.7554/eLife.78649 (PMC9586558; doi:10.7554/eLife.78649)
Supplement: Figure 4—figure supplement 1—source data 2. [file elife-78649-fig4-figsupp1-data2.pdf]

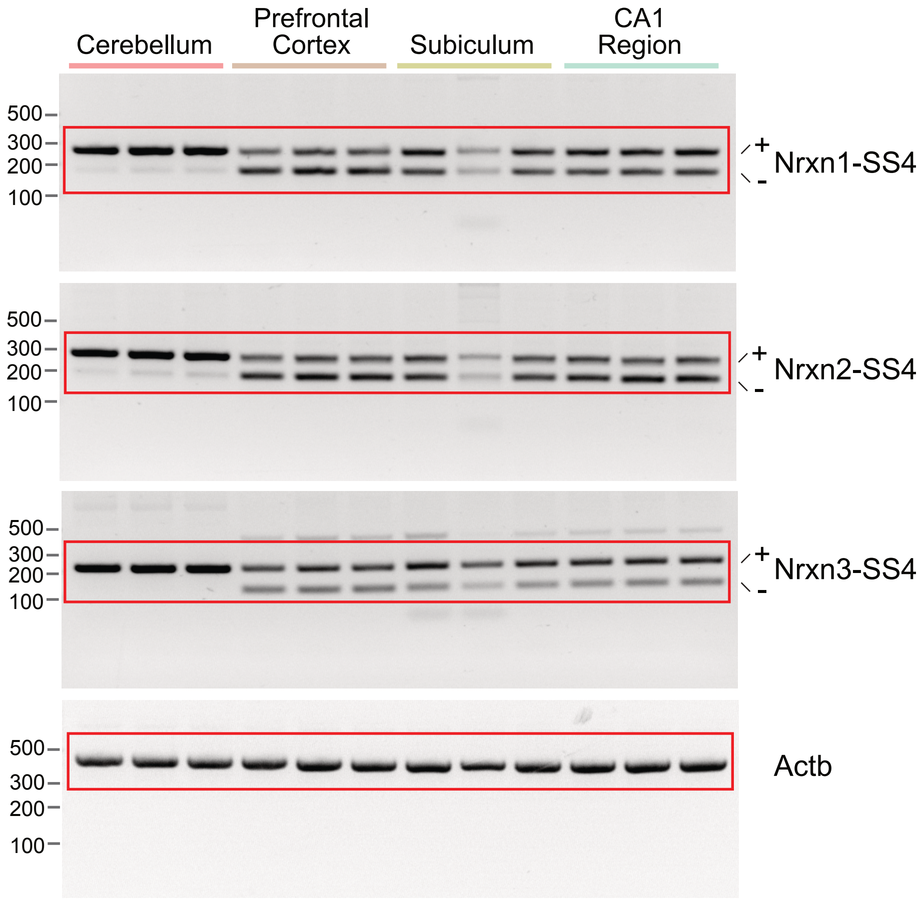

Figure supplement 3-source data 1. All Original full-sized gels correspond to Figure S3A (red box = sections shown).
